# The Value of Serum Adiponectin in Osteoporotic Women: Does Weight Have an Effect?

**DOI:** 10.1155/2021/5325813

**Published:** 2021-11-09

**Authors:** Ali B. Roomi, Wassan Nori, Saad H. Al-Badry

**Affiliations:** ^1^Ministry of Education, Directorate of Education Thi-Qar, Nasiriyah, Thi-Qar 64001, Iraq; ^2^Biochemistry and Biological Engineering Research Group, Scientific Research Center, Al-Ayen University, Nasiriyah, Thi-Qar 64001, Iraq; ^3^Department of Obstetrics and Gynecology, College of Medicine, Mustansiriyah University, Baghdad 10052, Iraq; ^4^College of Health and Medical Technology, Al-Ayen University, Nasiriyah, Thi-Qar 64001, Iraq

## Abstract

Osteoporosis (OP) has been observed to have a deleterious effect on postmenopausal women's life quality by increasing the risk of fragility fractures. The current research was adopted to verify the role of serum adiponectin, a cytokine released by adipose tissue, as a marker for OP across different body mass index groups, for a better understanding of fatty tissue role in OP. A case-control study recruited 210 eligible postmenopausal women and subgrouped into three groups based on their DEXA scan results: osteoporotic group, osteopenia group, and healthy controls; each includes 70 patients. Three datasets were collected: anthropometric, age, menopause duration, weight, height, body mass index (BMI), waist circumference, and fat percentage. Radiological examination estimated the bone mineral density (BMD) for the femoral neck and lumbar spines with their respective T-score. From blood, we measured alkaline phosphatase and calcium by a spectrophotometer and serum adiponectin, phosphate, CTX, and PICP by ELIZA. Total BMD, T-score, serum phosphate, and PICP were significantly higher among healthy controls. Serum adiponectin, CTX, and ALP scored higher levels among OP cases. A strong inverse relationship was proved between serum adiponectin and T-score in osteoporotic and osteopenia groups (−0.427, −0.301). A strong negative relationship was found between serum adiponectin and total BMD in healthy controls (−0.204). All correlations were statistically significant, *P* value <0.001. Serum adiponectin can be a valuable marker for reduced bone mineral density among the general populace, irrespective of the body mass index. Further research is warranted to explore therapeutic and preventive applications for this adipocytokine.

## 1. Introduction

Osteoporosis (OP) is a common metabolic age-related disease defined by skeleton remodeling and a reduced bone mineral density (BMD). It is expected to reach epidemic incidence in the coming century [1]. OP predisposes people, especially postmenopausal women (PMW), to fragility fractures with high morbidity and mortality; 24% of OP women have died because of their health consequences [2]. Osteoporosis is a silent multifactorial disease; some factors are unchangeable, such as age; elderly population has a 97% incidence rate, particularly those aged 75–84 years. In addition, OP has a particular predilection for PMW. Other nonmodifiable factors include multiparous women, early menopausal onset, and long menopause duration [1, 3]. One of the modifiable risk factors for OP is the body mass index (BMI); body weight claimed to be a key determinant factor in adulthood BMD and later on OP development risk [4]. Obesity increases the strain on the bones, increasing their density. Conversely, increased marrow adiposity impairs bone integrity, contributing to increased fracture risk. Considering the global obesity crisis and increasing life expectancy, osteoporotic fractures in obese older PMW and shorter recovery periods are expected [5, 6].

Adipocytes are cells found in the bone marrow besides the viscera and intestine. They have a pivotal role in skeletal integrity as they govern the development of stromal cells into osteoblasts, which create bones. In addition, they secrete particular mediators known as “adipokines,” such as adiponectin. Adipocytes rise in specific illnesses such as OP and obesity, and they have been related to bone strength deterioration, leading to higher fracture risk [7]. Estrogen is certainly a modulator of marrow adiposity; previous research stated that estrogen negatively influences adipocytes, and estrogen supplementation for two weeks reduces their number by 5%. As a result, there is a rising interest in bone marrow adipocytes as a targeted therapy for osteoporosis [8]. In addition, adiponectin receptors were described in osteoblasts, implying their role in bone formation by promoting proliferation and differentiation of osteoblast and inhibiting osteoclasts activity [9].

Studies linked adiponectin with an adverse effect on bone mass through intensive bone resorption, while others described a correlation between adiponectin plasma levels and BMD, highlighting its role as a predictor of osteoporotic changes [10]. The correlations between OP and BMI were investigated by earlier research; still, they gave inconsistent and sometimes contradicting results. Serum adiponectin levels were examined by other researchers, showing a negative correlation between visceral fat and BMI. Its levels were diverse among individuals with comparable BMIs, following age, sex, and hormonal influences [11]. The exact role of adiponectin in OP disease is not well understood [12].

Since OP is better prevented than treated, many researchers pursue serum biomarkers for earlier diagnoses to improve outcomes [3]. However, the relation of BMI, serum adiponectin to OP among the Iraqi populace, is scarce. This study primarily aimed to examine the levels of serum adiponectin in different BMI (as an anthropometric parameter) with BMD. The secondary aim was to verify serum adiponectin correlations between body mass index, BMD, and T-score for menopausal women with and without OP.

## 2. Patients and Methods

A descriptive case-control study taken from May 2020 to June 2021 recruited 210 postmenopausal women. All attended the geriatric clinic in our University Hospital and subdivided into 3 groups based on the DEXA scan results. The osteoporotic group (OP) included 70/210 osteoporotic postmenopausal females with OP changes, the osteopenic group (OPN) included 70/210 postmenopausal females, and the healthy controls group (CG) included 70/210 postmenopausal females. The study protocol was outlined to the participants, and written informed consent was sought after the Institute Ethical Committees approved it. Samples size was measured by the sample size equation. The number of samples required for the study was found.

(1) $$\begin{array}{c} n=\frac{Z^{2}*P\left(1-P\right)}{d^{2}}, \end{array}$$

where *n* represents the sample size, *Z* represents the *Z* statistic for a level of confidence, *P* represents the expected prevalence or proportion, and *d* represents the precision (in proportion of one; *d* = 0.001).

We enrolled women in menopause aged 50 years and up. Menopause was defined by the absence of menses for more than a year. With a detailed medical and gynaecological history, exclusion was made to women with renal, liver, endocrine, inflammation, whether acute or chronic, dietary, or physical activities disturbance in any of the participants, impairing the bone metabolism. Participants with disorders or risk factors for OP and those taking medications that impair the bone metabolism were omitted from the study. By the end of our assessment, we finally excluded cases who were underweight and morbidly obese (BMI (<18.5 and >34.9 kg/m^2^)) to decrease the risk of bias.

Besides the participant's age and menopause duration, other anthropometric readings were done twice in a barefoot standing pose, and we used the sum of two measures, including weight, height, BMI, waist circumference, and fat percentage. Weight in kilograms was used to measure the body mass index. Kilograms were divided by height squared (in meters); BMI = weight (kg) divided by the square of height (m^2^). Calculation of fat% score was done based on the following equation:

(2) $$\begin{array}{c} \text{Fat percentage}=\left[1.20\times \text{body mass index}-0.23\left(\text{age}\right)-10.8\left(0\text{ for female}\right)-5.4\right]. \end{array}$$

After many natural breaths, the WC was estimated at a plane parallel to the ground, at the midaxillary line, midway between the tip of the iliac crest and the lower border of the last perceptible rib. Cutoff values for Asians were used to analyze the data (80 cm in women).

  Radiological examination estimated lumbar spine and femoral neck BMD and their respective T-score
  After one night fast, blood was aspirated, centrifuged, and sera divided into two; the first part was used to measure alkaline phosphatase and calcium by the spectrophotometer. The rest of the serum was stored frozen at –20°C for later estimation of serum adiponectin by enzyme-linked immunosorbent assay (ELISA), phosphate, CTX, serum human carboxyterminal propeptide of type I procollagen, a marker for bone degradation, and (PICP) serum procollagen I carboxyterminal propeptide, a marker for bone formation.

## 3. Statistics

The Shapiro–Wilk test defined the data normality, and analysis was made by SPSS software program version 22.0 (SPSS Inc., Chicago, IL, USA). Data expression was as mean ± standard deviation. The one-way ANOVA test compared the groups' quantitative variables. Pearson's correlation coefficient tested the correlation between serum adiponectin versus BMI, total BMD, and T-score. One-way ANOVA test compared the serum adiponectin in the three study subgroups. Significant was set at *P* value <0.001 for all tests.

## 4. Modeling Results

To 210 postmenopausal women, a descriptive case-control study was performed.  Table 1 provides the essential criteria of the three groups under study. Age, menopause duration, fat%, waist circumference, BMI, and serum calcium failed to have statistical significance among the groups. Total BMD, including lumbar and femoral, T-score, including lumbar and femoral, serum phosphate, and PICP were significantly higher among CG. Serum adiponectin, CTX, and ALP scored higher levels among OP cases as *P* < 0.001.

In Table 2, serum adiponectin levels were compared to the BMI as an indicator of obesity. The main subgroup number was 69 samples instead of 70, where one sample was excluded because it was outside the study, subgroups, it had BMI (35.0–39.9 kg/m2). The subgroups were divided as follows:

  BMI (18.5–24.9 kg/m^2^): 7 samples
  BMI (25.0–29.9 kg/m^2^): 8 samples
  BMI (30.0–34.9 kg/m^2^): 8 samples

Serum adiponectin scored the highest levels in obese women (BMI of 30–34.9 kg/m^2^) for all the three groups, 14.47 ± 1.88 *μ*g/ml, followed by 9.47 ± 2.46 and 6.47 ± 1.47 *μ*g/ml for the overweight and healthy weight women (BMI 25.0–29.9 and 18.5–24.9 kg/m^2^, respectively). In summary, all the three groups' adiponectin concentrations increased with the increasing BMI group, and that for each BMI group, they were the consistently highest in the OP group. All differences were statistically significant, showing the positive association of serum adiponectin with BMI irrespective of the OP risk, *P* value <0.001.

Table 3 provides the relationship of serum adiponectin with the body mass index, BMD, and T-score in the three groups. A strong inverse correlation was observed between serum adiponectin and T-score in the OP and OPN groups (−0.427 and −0.301). A strong negative relationship was found between serum adiponectin and total BMD in healthy CG (−0.204). A modest positive relationship was proved between serum adiponectin with BMI in the OP and OPN subgroups (0.387, 0.201). All correlation was statistically significant, with a *P* value <0.001.

## 5. Discussion

Serum adiponectin was linked to the BMI with a positive correlation in OP and OPN; conversely, it showed a negative correlation with T-score. A strong negative relationship was confirmed between serum adiponectin and total BMD in healthy CG. Many debates exist on the effect of BMI on bone strength; some researchers confirm no association between BMI and OP, and others declared a significant correlation. Others claimed that the high BMI prevents OP by compensating for the detrimental impact of hypoestrogenic on BMD after menopause [13, 14]. Raising BMI improves bone density as mechanical loading of the bone increases. Higher adipose tissue reflected by increased BMI serves as an estrogen supply for women in menopause, and it seems to suppress osteoclast bone resorption. Interestingly, this defensive action will be missed if BMI surged into obesity, which explains the controversy in earlier research [9].

Effects of obesity on bone structure were investigated by Ibrahim et al. in a comparative study of obese Egyptian women in menopause to verify the impact of body mass index on BMD. The authors measured serum leptin and urinary C-terminal telopeptide at type 1 collagen level. They declared high serum CTX levels and low hip and spine BMD in obese postmenopausal women compared to healthy weight women [15]. The role of obesity was further cleared in Sharma et al.' study. They evaluated the impact of visceral fat as an inverse determinant for bone strength in women with OP, involving 76 participants and overweight PMW. They assessed bone turnover markers in both groups: total BMD, serum vitamin D, and parathyroid hormone levels. The authors compared visceral fat and BMI, showing the highest visceral fat values in PMW linked with low CTX, 25 (OH) D, and high BMD. All relations were statistically meaningful. The positive relationship of visceral fat with BMD, after BMI correction, showed a negative prediction of BMD *β* = 0.368, *P* < 0.05. Visceral fat is the most potent independent factor of BMD and bone turnover markers among all adiposity parameters. The study recommended using visceral fat and not BMI to predict bone strength in obese postmenopausal women. The estimated BMI may not represent the actual fat effect because of the diversity of fat distribution across the populace, especially in restricted areas' BMI range value [16].

Waist circumference, a commonly used parameter for visceral fat in clinical studies, showed no contribution to the critical visceral fat-based assessment of these bones. In the current study, serum adiponectin values were significantly high in OP group women and were associated with the lowest T-scores and total and lumbar BMD, across the three groups. Correlation showed significant results upon correlating serum adiponectin versus T-scores, BMI, and BMD. Therefore, we think that serum adiponectin in OP women increases in reaction to increasing demands in that category; there will be increased synthesis and excretion to safeguard the bone from OP and OPN [17, 18].

In line with our results, Al-Osami et al. reported significantly higher serum adiponectin concentration among type-2 diabetes mellitus, osteoporotic postmenopausal women compared to nondiabetic OP women. Their study investigated the effect of poor insulin control on diabetic women and its correlation to serum adiponectin versus OP risk [19].

The impact of adipocytokines on osteoporosis-related fractures was evaluated in a retrospective study. Researchers gathered data over 25 years, along with 7 years of following postmenopausal women, to determine fractures risk. The highest serum adiponectin values were associated with a higher vertebral fracture rate; the hazard ratio was 1.18, *P* value 0.02. Higher serum leptin levels associate a low incident of long-bone fracture; the hazard ratio was 0.70, *P* value = 0.03. Both parameters were recommended as an independent risk factor for predicting fractures [20]. Many researchers suggest that adiposity and OP in overweight/obese women have a complicated relationship. The local associations and endocrine-based activity exerted by fatty tissue are accredited to alter bone strength and fracturing risk in older adults.

Jürimäe and Jürimäe [21] confirmed that serum adiponectin has an independent effect on BMD among perimenopausal women; higher levels were detected in PMW than in perimenopausal women. They suggested adiponectin to be the link between fat mass and BMD. Later studies by the same researchers highlighted the inverse relationship between serum adiponectin versus the total and areal BMD [22].

Among the study strengths, we analyzed many bone parameters: total BMD, lumbar BMD, femoral BMD, and their respective T-scores. Though serum adiponectin was linked to OP markers in earlier studies, especially for high-risk populations with medical comorbidities among elderly people, this study has addressed the relationship from a new perspective highlighting the serum adiponectin in the bone metabolism, implying its utility to measure BMD among elderly postmenopausal women with no medical comorbidities.

This study has limitations; being a single-centre study is one. Another point is its design. Since both exposure and result are measured simultaneously in cross-sectional studies, we cannot capture the long-term influence of adiponectin on bone remodelling and bone mineral density. Therefore, establishing a real cause-and-effect link is not feasible [23]. Because of the limited prediction findings associated with cross-sectional design study types, we recommend a case-control study design with longitudinal data collection to explain serum adiponectin association with OP with better predictive values.

## 6. Conclusion

Our analysis suggests that the serum adiponectin level serves as independent risk factors for reduced BMD among different bodyweight groups. Therefore, its levels may help predict osteoporotic fractures, which enable optimal OP prediction. Osteoporosis is best avoided than treated; having reliable markers for earlier diagnosis is vital to improving disease outcomes.

## Figures and Tables

**Table 1:** Baseline parameters of the studied groups.

Parameters	OP (*n* = 70) mean ± SD	OPN (*n* = 70) mean ± SD	CG (*n* = 70) mean ± SD	*P* value
Age (years)	60.21 ± 6.32	59.63 ± 6.21	60.96 ± 5.91	0.154^ns^
Menopause duration (years)	12.21 ± 4.6	13.01 ± 3.54	12.31 ± 3.32	0.141^ns^
Fat (%)	13.01 ± 2.06	12.91 ± 2.56	13.64 ± 2.97	0.136^ns^
Waist circumference (cm)	79.54 ± 8.31	77.21 ± 8.01	79.63 ± 7.45	0.122^ns^
BMI (kg/m^2^)	27.01 ± 2.78	26.64 ± 2.97	26.45 ± 2.34	0.183^ns^
Total BMD (g/cm^2^)	0.63 ± 0.17	0.82 ± 0.19	0.89 ± 0.20	<0.001^*∗*^
BMD (L2-L4) (g/cm^2^)	0.67 ± 0.21	0.82 ± 0.57	1.09 ± 0.11	<0.001^*∗*^
BMD F (g/cm^2^)	0.41 ± 0.20	0.52 ± 0.14	0.68 ± 0.15	<0.001^*∗*^
T-score (L1-L4)	−3.32 ± 0.90	−1.95 ± 0.56	0.93 ± 0.13	<0.001^*∗*^
T-score F	−3.61 ± 0.98	−1.89 ± 0.11	0.90 ± 0.21	<0.001^*∗*^
Calcium (mmol/L)	1.89 ± 0.49	1.90 ± 0.51	1.92 ± 0.52	0.015^ns^
Phosphate (mmol/L)	1.42 ± 0.42	1.59 ± 0.54	1.95 ± 0.78	<0.001^*∗*^
ALP (U/L)	92.48 ± 6.21	80.04 ± 7.54	78.57 ± 8.65	<0.001^*∗*^
CTX (ng/mL)	1.85 ± 0.68	0.97 ± 0.45	0.45 ± 0.12	<0.001^*∗*^
PICP (ng/mL)	17.47 ± 0.46	18.01 ± 0.54	19.41 ± 0.45	<0.001^*∗*^
Adiponectin (*μ*g/mL)	13.54 ± 2.54	8.21 ± 2.01	7.99 ± 2.45	<0.001^*∗*^

OP, osteoporosis group; OPN, osteopenia group; CG, control group; *n*, no. of subjects; BMI, body mass index; BMD, bone mineral density; L, lumbar; F, femoral neck; ALP, alkaline phosphatase; CTX, serum human C-terminal telopeptides of types I collagen; PICP, serum human carboxyterminal propeptide of type I procollagen. Data are means ± SD.

**Table 2:** Comparison of serum adiponectin (*μ*g/ml) in the studied groups.

Studied groups (*n* = 69)	BMI (kg/m^2^) (*n* = 23)	Adiponectin (*μ*g/ml) mean ± SD	*P* value
OP	BMI (18.5–24.9 kg/m^2^)	10.48 ± 1.97	<0.001^*∗*^
	BMI (25.0–29.9 kg/m^2^)	12.87 ± 1.45	
	BMI (30.0–34.9 kg/m^2^)	14.47 ± 1.88	

OPN	BMI (18.5–24.9 kg/m^2^)	7.01 ± 2.47	<0.001^*∗*^
	BMI (25.0–29.9 kg/m^2^)	7.98 ± 2.49	
	BMI (30.0–34.9 kg/m^2^)	9.47 ± 2.46	

CG	BMI (18.5–24.9 kg/m^2^)	4.47 ± 1.88	<0.001^*∗*^
	BMI (25.0–29.9 kg/m^2^)	5.14 ± 1.87	
	BMI (30.0–34.9 kg/m^2^)	6.47 ± 1.47	

BMI (18.5–24.9 kg/m^2^), healthy weight *n* = 7; BMI (25.0–29.9 kg/m^2^), overweight *n* = 8; BMI (30.0 to 34.9 kg/m^2^), obesity *n* = 8. ^*∗*^*P* value< 0.001 (indicates a significant). The total no. of the studied groups was 69 divided as BMI (18.5–24.9 kg/m^2^): 7 samples, BMI (25.0–29.9 kg/m^2^): 8 samples, BMI (30.0–34.9 kg/m^2^): 8 samples. ns, nonsignificant.

**Table 3:** The correlations of serum adiponectin (*μ*g/ml) versus body mass index, BMD, and T-score in the studied subgroups.

Studied groups	BMI		Total BMD		T-score	
	*r*	*P*	*r*	*P*	*r*	*P*
OP	0.387	<0.001^*∗*^	−0.034	<0.001^*∗*^	−0.427	<0.001^*∗*^
OPN	0.201	<0.001^*∗*^	−0.059	<0.001^*∗*^	−0.301	<0.001^*∗*^
CG	−0.025	<0.001^*∗*^	−0.204	<0.001^*∗*^	−0.021	<0.001^*∗*^

^*∗*^Correlation is a significant level at 0.001.

## Data Availability

The data used to support the findings of this study are available from the corresponding author upon request.
